# Characteristics of Selected Bioactive Compounds and Malting Parameters of Hemp (*Cannabis sativa* L.) Seeds and Malt

**DOI:** 10.3390/molecules29184345

**Published:** 2024-09-13

**Authors:** Marek Zdaniewicz, Robert Duliński, Krystyna Żuk-Gołaszewska, Tomasz Tarko

**Affiliations:** 1 Department of Fermentation Technology and Microbiology, Faculty of Food Technology, University of Agriculture in Krakow, Balicka Street 122, 30-149 Krakow, Poland; 2 Department of Biotechnology and General Food Technology, Faculty of Food Technology, University of Agriculture in Krakow, Balicka Street 122, 30-149 Krakow, Poland; robert.dulinski@urk.edu.pl; 3 Department of Agrotechnology and Agribusiness, Faculty of Agriculture and Forestry, University of Warmia and Mazury in Olsztyn, Oczapowskiego Street 8, 10-719 Olsztyn, Poland; kzg@uwm.edu.pl

**Keywords:** hemp (*Cannabis sativa* L.), malting, germination, bioactive substances, phytate, size exclusion chromatography

## Abstract

Hemp (*Cannabis sativa* L.) seeds are an interesting raw material for malting regarding its relatively high bioactive compounds concentration and proven advantageous properties in different food products and dietary supplements. In the first stage of the study, important seeds properties relevant to the malting process including moisture content, seed viability, and water absorption capacity were determined. However, a few parameters determining the seeds’ usability for malt preparation, such as germination ability and water sensitivity, are different in comparison to typical malting raw materials such as barley or wheat. However, they make it possible to obtain high-quality hemp malt. In the next stage of research, spectroscopic and chromatographic analyses, including measurements of antioxidant activity and protein separation by SEC-HPLC, were conducted. The results showed that the malting process improved the total antioxidant potential of hemp seeds by 15%, leading to an increase in the concentration of lower molecular weight proteins and oligopeptides—below molecular mass of 10 kDa—responsible for this high antioxidant activity. The processing of hemp seeds reduced the phytate content while increasing phosphate fractions with fewer phosphate groups, which may have a beneficial effect on nutritional value. These results suggest that malting hemp seeds needs optimalization of the process but can increase its nutritional value as a promising raw material in the food industry.

## 1. Introduction

The *Cannabis sativa* L. is one of the oldest species domesticated by humans. Nowadays, this species includes two botanical forms: *Cannabis sativa* subsp. *sativa* and *Cannabis sativa* subsp. *indica* (marijuana). It is widely cultivated around the world for its use in the production of fibre and paper (stems) [1]. Pharmaceutical raw materials include inflorescences used in the treatment of neurological diseases (tetrahydrocannabinol—THC and cannabidiol—CBD) [2,3,4]. Additionally, *Cannabis* seeds, due to their high nutritional value, constitute “functional food”, understood as food-containing substances or live microorganisms that have a possible health-enhancing or disease-preventing value, at a concentration that is both safe and sufficiently high [5]. They are a valuable source of protein, fatty acids, macro- and microelements, and bioactive compounds that enhance the diet, including vegetarian diets. Hemp protein isolate (HPI) is the most purified form, achieved through extraction techniques that provide chemical–physical treatment aimed at optimising protein sediment and removing non-protein fractions [6,7]. An additional potential benefit, in addition to adding hemp flour to flour from other crops to bake bread [8], is obtaining malt from hemp seeds. Malt has been gaining popularity in recent years as it is being used as a component in food products and dietary supplements [9]. Hemp seed malt, as an intermediate or raw material in the fermentation industry, may be a promising source of bioactive compounds. As shown by Leonard et al. [10] and Łopusiewicz et al. [11], the food fermentation process is an effective method for improving its nutritional and functional value through antioxidants, resulting in cell wall breakdown and the synthesis of various bioactive compounds [12].

In recent years, there has been increased interest in the effects of hemp on human health, particularly due to the potential anti-inflammatory, analgesic, anticonvulsant, and neuroprotective effects of cannabinoids [2]. However, there is little scientific research that thoroughly examines the chemical composition and potential health benefits of hemp seed malt. This is particularly intriguing because, in the case of various grains and pseudocereals, numerous studies have analysed the influence of germination on the chemical composition of the resulting malts. Moreover, it has been proven that malting improves the health and nutritional value of the raw materials used [9]. Therefore, a decision was made to address the issue of malting a relatively new raw material for the malting industry, namely hemp seeds.

Phytic acid (myo-inositol 1,2,3,4,5,6-hexaphosphate, InsP6) was chosen for analysis as an important antinutritional factor. It is defined by its ability to form complexes with proteins and minerals [13,14], and is also the main reservoir of phosphorus in plant tissues, present in food in the form of salt (phytates). In food products of plant origin, phytic acid levels range from an average of 0.29–1.05% in cereals, pseudocereals, and legume seeds, relatively high in sesame (approx. 5%), and higher in hemp (7–9%) [15]. However, one should not forget about the dual nature of this compound and its potential beneficial effects. It is important to note that phytate complexes and their derivatives may help prevent some gastrointestinal cancers by neutralising substrates for the Fenton reaction [16].

The aim of this work is to conduct a selective analysis of malting parameters and bioactive compounds of hemp (*Cannabis sativa* L.) seeds and malt, including the profile of proteins and antinutritional factors.

## 2. Results

### 2.1. Qualitative Characteristics of Hemp Seeds and Their Potential for Malting

The experiments conducted on other plant species have demonstrated that the qualitative characteristics of malts, including the content of their bioactive components, are largely related to the malting process and the parameters of its subsequent stages. In the case of barley malt, modification of individual stages of malt production affects, among other things, the enzymatic activity of the grain, the degree of its loosening, the content of B-glucans, and many other features [17,18]. Therefore, in the initial phase of assessing the quality of hemp malt, an analysis of important (for the malting process) qualitative characteristics of the raw material was performed. This included, among other factors, determining the moisture content (Table 1), which was found to be approximately 9%, a typical value for hemp. According to Sheichenko et al. [19], this moisture level can be used to produce hemp press cake, for example. Moreover, it is worth noting that excessive moisture would necessitate the quick processing of the material, primarily to avoid microbiological infections.

When considering the usability of grain, its 1000-seed weight is crucial, as a higher weight suggests better raw material quality. Gaybullaev et al. [20] focused on analysing the 1000-seed weight of several hemp varieties. The results, ranging from 15.9 g to 17.5 g, are significantly higher than the 11.76 g obtained in this work (Table 1). Therefore, this prompted an investigation to identify the reasons for the different results and the factors influencing the grain’s quality. In their studies on wheat, Shapowal and Mozharova [21] observed that the use of foliar fertilisation with a double dose of fertilisers containing chelated trace elements can increase the 1000-seed weight by up to approximately 3.4 g. In comparison, Khanal and Shah [22] demonstrated values up to 20 g for hemp. While analysing studies on the 1000-seed weight, it was observed that this value is often given without considering conversion to dry mass. Additionally, Sacilik et al. [23] pointed out a linear increase in the 1000-seed weight (from 15.3−16.9%) with increasing humidity (from 8.62% to 20.84%). Therefore, using the equation developed by Sacilik et al. [23], the mass of 1000 grains in this study can be calculated at approximately 15 g. Equally important in determining seed quality is a sieve analysis (gradation test). This involves sifting the material through sieves with different hole sizes. Unlike barley grain, hemp seeds required modification of this method by adding additional sieves with smaller holes. Due to the small size of hemp seeds (less than 2.5 mm), 1.5 mm and 1.8 mm sieves were added to the standard method. The aim was to assess the distribution of different grain sizes and to help select grain soaking parameters. This is crucial because the rate at which water is absorbed by the grain depends on its size [24]. Considering the results obtained, it can be observed that the seeds varied in size. The large number of seeds retained on 2.5 mm sieves (43.83%) and 1.8 mm sieves (36.53%) indicates a significant diversity in seed size. This diversity requires careful monitoring during soaking, as it may result in the premature germination in some seeds. Interestingly, different seed sizes may also explain the premature initiation of seed germination observed by Farinon et al. [9] in their malting samples. Premature germination was caused by smaller seeds reaching the critical moisture content enabling germination. According to the literature, achieving critical moisture content is necessary to initiate this process and varies for each plant species (e.g., 30% for corn) [25]. For the two tested hemp varieties, Secuieni Jubileu seeds and Futura 75 seeds, Farinon et al. [9] indicated critical water content values of 34% and 36%, respectively. This study represents the only literature known to the authors on hemp malt production. Notably, these levels were exceeded in this study during a six-hour soaking period (37.31%), resulting in the initiation of germination. Compared to barley, this value is significantly lower, but it is important to note the completely different chemical composition of these raw materials. The purpose of soaking is, among other things, to initiate the grain’s vital functions and facilitate the next stage, which is germination. To establish the potential number of grains capable of germination, an analysis of germination capacity was performed. This is one of the most important seed parameters [20]. Based on the result of 78.67% (Table 1), it can be demonstrated that approximately three-quarters of the seeds are capable of germination. However, compared to barley, where the germination capacity is approximately 97%, this result is quite low [26]. The number of germinating seeds also depends on their sensitivity to water. To check this sensitivity, a comparative test was carried out for barley with different amounts of water. The obtained grain sensitivity to water, measured at 58.02%, indicates greater sensitivity compared to many barley samples. However, this determination may be modified by considering the smaller seed size and thus reducing the water dosage, e.g., to 3 mL and 6 mL. It is also important to observe the seeds’ cross-section. The obtained results (Table 1) show that the vast majority (92.97%) have the expected appearance. This analysis allows for the identification of an excessive starch and protein granules compaction in the barley and barley malt endosperm (steeliness) [27]. However, this relationship has not been proven in the case of hemp due to its different chemical composition.

### 2.2. Protein Profile

**Figure 1:**
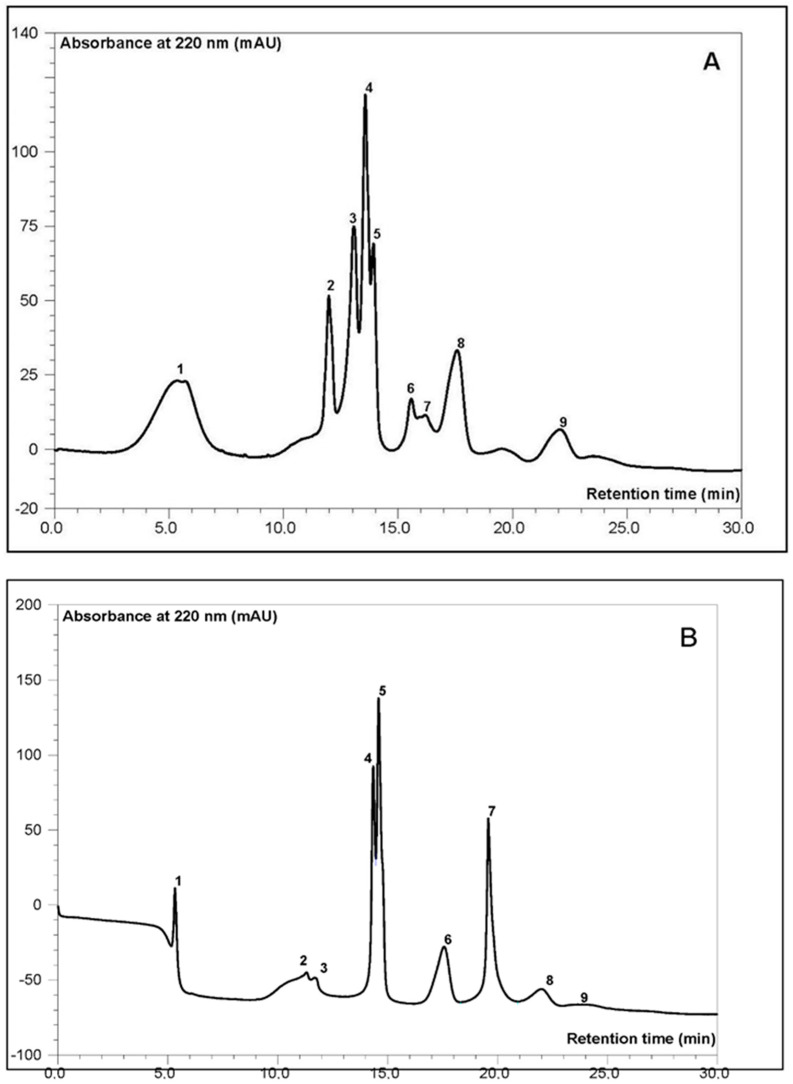
Example of SEC-HPLC profile from raw (**A**) and malted hemp seed (**B**) protein isolate. Peak nr. 1–2: high molecular weight proteins, 3–5: edestins and albumins, 6–9: low molecular weight proteins and oligopeptides.

**Figure 2:**
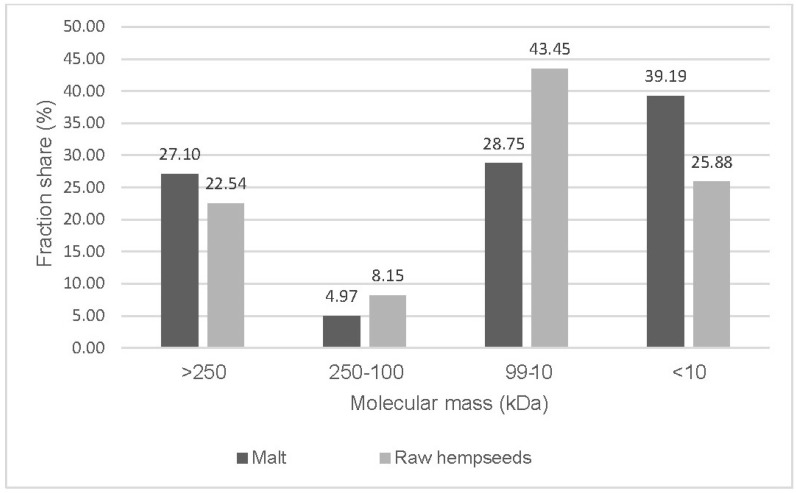
Distribution of the estimated molecular mass (kDa) fractions of the proteins.

A TSK-Gel GMPWXl column with a wide fractionation range (1000–10 kDa) was used to separate protein fractions, allowing for the monitoring of both high molecular weight proteins and peptides below 10 kDa (Figure 1 and Figure 2).

Significant changes in the polypeptide profile were noted, especially when compared to an additional reference object, Futura hemp protein isolate (unpublished data). Generally, the SEC-HPLC profile obtained for malt is characterised by an evolution towards proteins with a lower molecular weight. This is most likely related to the activation of endogenous proteolytic enzymes, resulting in an increase in protein concentration and the appearance of new protein peaks in this low molecular weight range, likely oligopeptides. The profile identified a band typical for hemp seeds, correlating with the band characterised in previous works [28] with a retention time range of 12–15 min (peaks no. 3–5), representing edestin and albumin proteins in the molecular weight spectrum of 15–70 kDa, including both basic and acidic subunits of heterologous nature. In malt, in addition to the bands mentioned above, which evolve during the process towards proteins and peptides with lower molecular weights (Figure 1A,B), such a phenomenon was also noted during the analysis of malt and buckwheat wort in the work of Duliński et al. [29]. In the work of Mamone et al. [28], the authors identified a number of proteins or peptides with a molecular weight lower than 10 kDa in the electrophoretic image, which is also confirmed by our observations. The SEC-HPLC spectrum shows several bands (retention time 22–25 min) with a dominant area of approximately 39% in the total spectrum (Figure 1B). It is worth noting that many oligopeptides identified and characterised in this molecular weight range—as suggested by the research of Grigih et al.—may exhibit antiarrhythmic, hypotensive, and anticholesterolemic properties [30].

### 2.3. Antioxidant Activity

There is a wide spectrum of tests used to analyse the antioxidant parameters of food products, and in this study, one of the most common tests was used, inhibiting the spectral properties of the DPPH radical.

The total scope of inhibition of the DPPH radical by the summed fraction of the tested materials shows a much higher degree of inhibition of the DPPH radical by malted seeds (78.57%) compared to raw seeds (63.60%).

In the next stage, the activity of individual protein fractions from SEC-HPLC, fractionated in 1 min intervals and then concentrated using a concentrator, was presented. In the determined fractions, the highest percentage inhibition of the DPPH cation radical was demonstrated by fraction no. 6 (estimated molecular weight above 250 kDa according to SEC-HPLC), identified both in raw seeds and malt.

**Figure 3:**
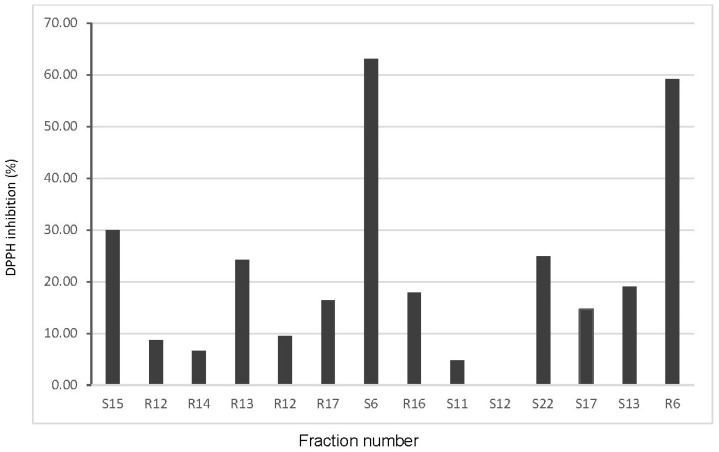
DPPH radical scavenging activity (%) of raw (R) and malted hemp seed (S) protein fractions separated by SEC-HPLC. The fraction number is directly correlated with the elution time in minutes on the HPSEC column.

The next fractions showing significant antioxidant activity were those collected at 13 and 16–17 min. Analogies between raw seeds and malt can be observed here as well. However, a factor influencing the greater potential of malt, according to these determinations, is the presence of oligopeptides (MW < 10 kDa) identified in the 20–22 min time interval of SEC-HPLC separation (Figure 1B), with a DPPH inhibition level of 25% (Figure 3). The high antioxidant activity of enzymatically digested (trypsin, pepsin, alcalase) hemp protein isolates fractionated on Sephadex G-25 was confirmed in the research of Tang et al. in a broad spectrum of antioxidant tests [31].

According to the results obtained, malted hemp seeds are characterised by a higher antioxidant potential compared to raw seeds. This is most likely the result of an increase in polyphenol content and a high deposition of tocopherols in the seeds, which possess antioxidant properties documented in numerous studies [32,33]. During the malting of hemp seeds, enzyme activation, oxidative stress, and metabolic changes trigger the production of these compounds, whose level increases as the seeds prepare to protect themselves from environmental stress and sustain growth during germination. During the process, lipid mobilization occurs to provide energy for the growing seedling, and tocopherols help protect these lipids—the level of which is especially high in hemp seeds—from oxidation. Consequently, malted hemp seeds could have higher concentrations of these beneficial compounds compared to their non-malted counterparts.

According to the literature, the most abundant phenolic amide in both raw and malted seeds is *N*-*trans*-caffeoyltyramine [2,9]. Because it is located in the hemp seed husk, the malting process, particularly the high-temperature drying stage, contributes to changes in the seed structure, which stimulates the release of the mentioned phenolic amide. Furthermore, data from the literature indicate a lower amount of *para*-hydroxybenzoic acid in malted seeds [2]. Since it is a phenolic acid, it can be treated as a component for lignanamide biosynthesis, which increases both the amount of phenolic compounds and antioxidant activity, while decreasing the acid concentration. Although lignanamides are not typically associated with the malting process, the conditions created by malting (such as enzymatic activity, changes in pH, and the release of certain compounds), particularly the germination phase, could potentially support the biosynthesis of these secondary metabolites.

### 2.4. Phytic Acid and Inositol Phosphates

It should be emphasised that the methodology for analysing this group of compounds, defined as phytates, relies predominantly on indirect analytical techniques, as indicated by the available literature. These procedures involve determining phosphorus or its derivatives using colorimetric methods applied to extracts after complex precipitation with iron ions. On this basis, taking into account the available data on the P:InsP6 ratio (factor 3.55 or 29.8%), the content of this compound is calculated [34]. Alternatively, a commercial kit, based on the measurement of phosphorus released in a phytase-catalysed reaction after reaction with a molybdate reagent, can be used [35]. Most of these are indirect methods and should be treated as indicative, though they appear as reference points in relatively new works from reputable publishers [36].

The existing challenges in precise determination using modern techniques such as HPAEC (high performance anion-exchange chromatography) with off-column derivatisation and UV-Vis detection or conductivity detection with anion suppression mode (CD-AMMS) may also result from the high lipid content. During the determination of inositol phosphates, the high lipid content significantly complicates the extraction of phytate, necessitating degreasing and the elimination of interfering factors from the extract to facilitate accurate analysis of this group of compounds.

To analyse myo-inositol phosphate profiles, one of the newer available analytical methods was used, with high-performance anion exchange chromatography and a Carbo-Pack PA-1 column [37]. The analysis of this antinutritional factor was performed using the colorimetric method with the Wade reagent.

**Figure 4:**
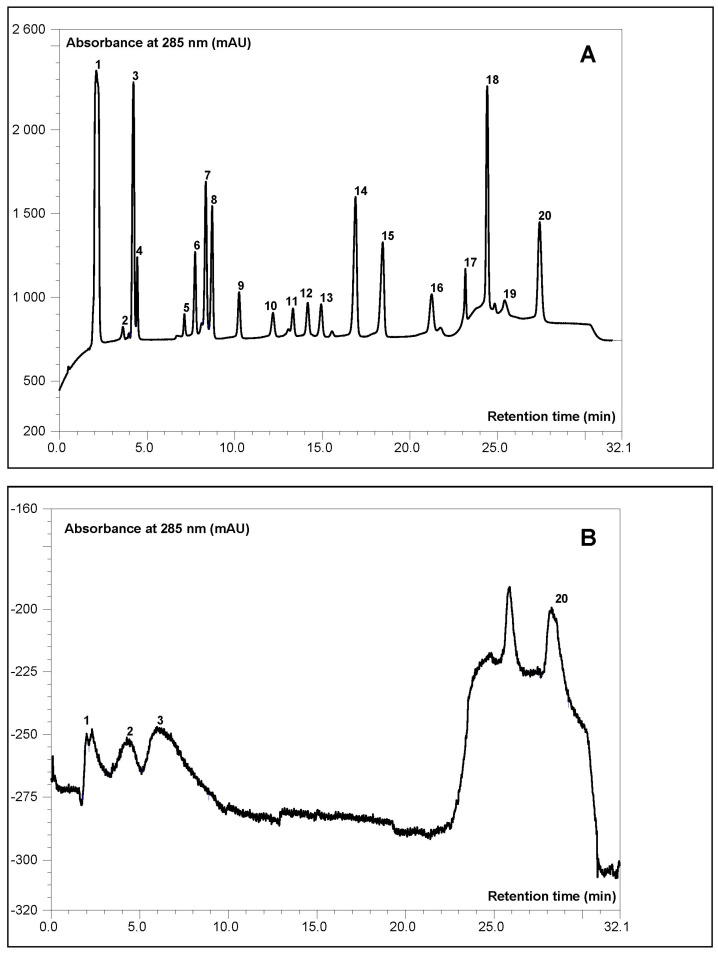
Exemplary high-performance anion-exchange chromatography (HPAEC) chromatogram of inositol phosphate profile of: (**A**) standard, thermal hydrolysate of InsP6 (**B**) raw hemp seed seeds. 1—Phosphate; 2—InsP1; 3—Ins(2,4,6)P3; 3—InsP3; 4—Ins(2,4,5); (1,4,5)P3; 5—InsP3; 6—InsP4; 7—InsP4; 8—InsP4; 9—InsP4; 10—InsP4; 11—Ins(2,4,5,6)P4; 12—Ins(1,2,3,6)P4; 13—Ins(1,2,3,4)P4; 14—InsP4; 15–18—InsP5; 19—I ns(1,2,4,5,6)P5; 20—phytate.

The base profile for hemp seeds not subjected to microbiological treatment indicates a dominant presence of hexaphosphate (26%) and lower inositol phosphates (InsP1–2) (69%) (Figure 4). The malting process contributes to slight changes in the distribution of lower phosphate profiles and a reduction in the share of InsP6, where a decrease (approx. 19%) was noted, followed by an increased share of phosphates with 1–2 phosphate groups (over 55%) and InsP3 (6.9%) of unidentified configuration of phosphate residues in the polyalcohol ring. Phosphates with an intermediate number of phosphate groups (4–5) were not identified in the spectrum.

The obtained spectrum for malted seeds may result from the transformation of a certain fraction of lower forms of inositols into a pool of phosphorus and myo-inositol itself, components crucial for activating life processes in the soaked and pre-sprouted grains [38,39].

The phytic acid content in raw hemp seeds determined by the colorimetric method with the Wade reagent is 56.25 mg, which is 10–25% lower than data from other sources (61.2–76.7 mg/g) [34,40,41,42]. This may result from genetic differences, phenotypic changes in varieties, or certain differences in the process of material extraction and degreasing [35,41].

The treatment process related to the malting of *Cannabis sativa* seeds results in a nearly 19% reduction in the content of this antinutritional factor (Table 2). These observations are consistent with the data presented by Farinon et al. [9], among others.

According to the literature, the malting process contributes to reducing the content of antinutritional factors contained in hemp seeds [42,43]. One of these factors is phytic acid, which naturally occurs in plants in the form of salts known as phytates, and acts as a store for phosphorus deposited in food products [14]. However, due to its ability to form insoluble complexes with proteins and minerals, it disrupts their absorption by the human body.

The malting process, particularly the sprouting and soaking stages, stimulates endogenous phytases activity [44]. These phosphorolytic enzymes are relatively well-characterised in microbial sources [45]. This also applies to some crop plant species, especially cereals [46]. However, in the case of hemp seeds, their activity and selectivity remain unrecognised.

## 3. Materials and Methods

### 3.1. Materials

Hemp seeds of the Henola variety were used. The seeds were cultivated in Poland and supplied for research by Hemp Farm Poland (Krakow, Poland).

### 3.2. Seed Analysis

#### 3.2.1. Weight of 1000 Seeds

The weight of 1000 seeds was determined by weighing two approximately 40 g portions of seeds. After removing damaged seeds and foreign bodies, the number of seeds was estimated according to the following method: 3.4 Thousand Corn Weight (Analytica EBC, European Brewery Convention, 1998) [47].

#### 3.2.2. Moisture Content

Seed moisture was analysed by grinding seeds in a laboratory mill for 12 s. Then, 4 g of seeds from each sample were taken and placed in a MAC 50 moisture analyser (RADWAG, Radom, Poland).

#### 3.2.3. Seed Viability

The tetrazolium test was used to assess seed viability. In this method, 100 seeds were cut and placed in a 50 mL beaker with a 1% TTC (2,3,5-Triphenyl Tetrazolium Chloride) solution. Staining was performed in a dark, incubated environment for 30 min. After staining, the seeds were placed in Petri dishes, and seeds with stained (red) embryos were counted.

#### 3.2.4. Water Sensitivity

Seed sensitivity to water was determined by placing an appropriate portion of barley (100 seeds) in Petri dishes, followed by soaking the seeds in the analysed water samples (4 mL and 8 mL). The number of germinated seeds was determined after 48 h.

#### 3.2.5. Water Uptake Capacity (Swelling Ability)

The water uptake capacity of the seeds (W2) was tested by placing 100 g of hemp seeds in a pot and soaking it in 500 mL of water. After a predefined soaking time (6 h), the sample was weighed (after draining). The seed soaking capacity was calculated from the obtained masses using the following formula:

W2 = 100 − M1·(100 − W1)/M2(1)

where M1 and M2 are the sample masses (g) prior to and after soaking, respectively, and W1 and W2 are the water contents before and after soaking (%), respectively.

#### 3.2.6. Grading by Seed Size

Grading by seed size was analysed by placing a 100 g seed sample on the frame of a sorting machine with appropriately located sieves (1.5, 1.8, 2.2, 2.5, and 2.8 mm). The sorting machine was run for 5 min. Afterwards, useful and useless impurities were removed from each sieve and transferred onto the bottom cover. The purified seed fraction and the residue on the bottom were weighed separately according to the modified (adding 1.5 mm and 1.8 mm sieves) method: 3.11.1 Sieving Test (Analytica EBC, European Brewery Convention, 1998) [47].

#### 3.2.7. Cross-Cut Observation (Endosperm Property)

The endosperm was observed by cutting seeds with a steel scalpel and was evaluated either as “appropriate” or “inappropriate” (abnormal).

### 3.3. Malting Procedure

The process of malting hemp seeds was carried out under laboratory conditions at the Department of Fermentation Technology and Microbiology of the University of Agriculture in Krakow. The process consisted of three main stages: soaking the grain, germination, and drying. The total soaking time lasted six hours and consisted of nine water and nine air cycles. For this purpose, 100 g of seeds were soaked in 500 mL of water at 19 °C for 30 min, then the water was removed and the seeds were left out for five minutes while being constantly shaken. After completing the required number of soaking cycles, the samples were weighed and transferred for germination. During germination, the soaked seeds were placed at a temperature of 20 °C. Every 24 h, their surface was sprinkled, and the entire batch was mixed. The germinated seeds were dried at 50 °C for 5 h to the desired moisture level (below 4% m/m).

### 3.4. Spectrophotometric Analyses

#### 3.4.1. Preparation of Extracts for Antioxidant Activity Determination 

For the analyses of antioxidant activity, 1 g of each sample was extracted with 80% aqueous ethanol (1:10, *w*/*v*) in the dark for four hours. The samples were then centrifuged at 4500× *g* (MPW-320 centrifuge; MPW Med Instruments, Warsaw, Poland) for 20 min at 4 °C. The supernatant was collected and used for the TPC and total antioxidant capacity determination.

Antioxidant activity measurements were made using a methanolic (80%) DPPH solution at a concentration of 0.1 mmol/dm^3^. For the measurements, 0.2 mL of extracts and 2.8 mL of DPPH were used. Additionally, reference tests were performed using 0.2 mL of 50% acetone instead of a sample, and Trolox (a synthetic derivative of vitamin E) equivalents. Samples were placed in the dark, and measurements were taken after 30 min at a wavelength of 516 nm using 80% methanol as a reference. Hydroxyl radical scavenging activity (%) was calculated using the formula: ((A0 − A1)/A0) × 100, where A0 is the absorbance of the control and A1 is the absorbance of the sample. The results are also expressed as μg Trolox Equivalent per gram of sample (μg Trolox/g).

#### 3.4.2. SEC-HPLC Separation of Hemp Seed Proteins

Hemp seed proteins (HP) were separated by SEC-HPLC on a Dionex Ultimate 3000 system (Thermo-Dionex, Sunnyvale, CA, USA). Freeze-dried HP was dissolved in solvent A (double-distilled water) at a concentration of 100 mg/mL. A volume of 100 μL (filtered through 0.45 μm membrane disks) was injected onto a TSK-Gel GMPWXl (4 × 375 mm) semi-preparative column (Tosoh Bioscience, Tokyo, Japan). Fractions were eluted from the column at a flow rate of 1 mL/min using an isocratic mode (solvent A: 0.01 M phosphate buffer, pH 7.00) over 30 min. Elution of protein fractions was monitored by absorbance at 220 nm. Fractions were collected using an automated fraction collector model 2128 (Bio-Rad, Hercules, CA, USA) every minute and pooled into fractions according to elution time. The pooled and combined fractions (F1–F8) from four consecutive runs were evaporated using a Kamush evaporator (Lipopharm, Zblewo, Poland) and stored at −20 °C until required for further analysis. Each of the freeze-dried fractions was assayed for antioxidant activity using the DPPH scavenging test.

### 3.5. Phytate Analysis

#### Semi-Qualitative Analysis of the Inositol Phosphate Profile

Extraction of inositol phosphates from samples was conducted according to Duliński et al. [29]. A 2 g sample of material was extracted in 20 mL of 0.5 mol∙L^−1^ hydrochloric acid at room temperature for two hours. Extracts were centrifuged for 30 min at 2038× *g* and filtered. Low-pressure ion exchange liquid chromatography was performed to separate myo-inositol phosphates from the extract. Chromatography columns were filled with 2 g of analytical grade, 8% crosslinked anion-exchanger (37–74 µm, chloride form, Bio-Rad Laboratories, Hercules, CA, USA) and conditioned with 10 mL of deionised water. Next, 15 mL of the extract was passed through the column, and the column was rinsed with 10 mL of deionized water. Elution of myo-inositol phosphates was performed using 20 mL of 2 mol∙L^−1^ hydrochloric acid. Eluates were evaporated in a water bath preheated to 40 °C, redissolved in 5 mL of deionized water, frozen at −18 °C, and stored for HPLC analysis.

### 3.6. HPLC Analysis

The profile of the isomers of myo-inositol phosphates was analysed using a high-pressure anion-exchange chromatography (HPAEC) system [37] consisting of a model WPS-3000SL autosampler, a thermostatted column compartment (TCC-3000), a LPG-3400A gradient pump, an ISO-3000 isocratic pump, and a PDA-3000 photodiode array detector (all from Dionex, Sunnyvale, CA, USA). Analyses were performed on a Dionex CarboPac^TM^ PA1 guard column (Thermo-Dionex, Sunnyvale, CA, USA) (50 mm × 4 mm i.d., 10 µm) and a CarboPac^TM^ PA1 analytical column (Thermo-Dionex) (250 mm × 4 mm i.d., 10 µm). Dionex Chromeleon software v. 6.80 was used for control of the HPLC and collection of data.

Two mobile phases were used: (A) 1.5 mol L^−1^ methanesulfonic acid and (B) deionized water (18 µS conductivity). The separation of InsP2–InsP6 was performed by using gradient elution according to the parameters described below at a column temperature of 30 °C (Table 3).

The eluate was monitored at 285 nm after post-column reaction with 0.1% Fe(NO_3_)_3_·9H_2_O and 2% HClO_4_ in water (*w*/*v*) and derivatization of InsP, forming complexes with Ferric ions. The flow rates of eluents and post-column reaction solution were 1.0 and 0.65 mL min^−1^, respectively, and the injection volume was 50 µL. Before injection into the chromatographic column, samples were filtered through a syringe filter (0.45 μm). A mixing tee and a knitted reaction coil (750 µL, 0.019 mm i.d. × 0.050 mm o.d., from VICI-Valco (Houston, TX, USA) was used. Every sample was analysed in three consecutive runs.

A reference sample for identifying peaks was prepared by dissolving 2.3 g of sodium phytate in 50 mL of deionized water and adjusting the pH to 4.0 by 2 M HCl. The solution was autoclaved for 40 min at 121 °C under 1 atm. The elution sequence of different isomers was established according to the work of Blaabjerg et al. [37] using the appropriate standard solutions, i.e., sodium phytate (InsP_6_), Ins(1,2,4,5,6)P_5_, Ins(1,4,5,6)P_4_, Ins(1,3,4,5)P_4_, Ins(1,4,5)P_3_, Ins(1,3,4)P_3_, and myo-inositol 2-monophosphate (all purchased from Sigma-Aldrich, Saint Louis, MO, USA).

### 3.7. Colorimetric Analysis Method Using the Wade Reagent

From the fraction purified according to the procedure presented above, 300 μL of the sample was taken into 1.5 mL Eppendorf tubes, and 100 μL of Wade reagent was added. The solution was mixed using a vortex mixer for approximately 5 s, then centrifuged for 6 min at 12,000× *g* (MPW 320 centrifuge, MPW Med Instruments, Warsaw, Poland). Subsequently, 300 μL of the supernatant was collected into a well on a 96-well microplate (Becton Dickinson, Franklin Lakes, NJ, USA). The absorbance was read on a microplate reader at a wavelength of 490 nm. Quantitative analysis was performed in the microplate manager programme based on the prepared standard curve. Phytic acid standards underwent the same treatment as the obtained eluates. Absorbance readings were recorded on a microplate reader at a wavelength of 490 nm, and quantitative analysis performed using the Elisa Sunrise™ microplate reader (Tecan, Männedorf, Switzerland).

### 3.8. Statistical Analysis

Experimental data were subjected to a one-way analysis of variance (ANOVA) to detect significant differences among means, and results are expressed as mean ± standard deviation (SD). Differences among means were checked by the Tukey test at *p* < 0.05 using Statistica for Windows, version 13.0 (StatSoft Inc., Tulsa, OK, USA) statistical software.

## 4. Conclusions

Hemp (*Cannabis sativa* L.) seeds have a high potential for malt production. More than 3/4 of the seeds can be transformed into high-quality malt. However, the malting parameters of seeds differ significantly from the standard malting raw material, which is barley. Therefore, before starting the malting process, it is necessary to consider, among other things, the different size of the grains and therefore it is recommended to sort them beforehand. This operation will ensure the same level of water absorption in the entire batch of grains during steeping. Obtaining a value of 37% moisture of the seeds is sufficient to initiation of germination. Moreover, it is worth considering the high sensitivity of the grain to water during this stage (significantly higher compared to barley).

Comparative analysis of hemp malt and raw seeds indicated significant differences between the analysed materials. During analysis of the protein profile using HPSEC, several fractions with varied molecular weights in the range of 250–10 kDa were identified. Proteins with an average molecular weight predominated in raw seeds, while in malt, their level was below 10 kDa. A comparison of antioxidant parameters and the protein profile indicates the decisive role of lower molecular weight fractions in shaping the antioxidant potential of preparations. This evolution is most likely due to changes caused by metabolic processes initiated in the pre-sprouted grain and the release of proteolytic enzyme activity.

The study also attempted to analyse the profile of phytate, considered an antinutritional factor, and inositol phosphates, which are thought to have a potentially dual nature, using the HPLC technique. The malting process confirmed the beneficial effect of the treatment in reducing InsP6 content while simultaneously altering the distribution of lower inositol phosphate fractions, which are dominant in the spectrum, with one to three phosphate groups. 

In the next stage of the research, efforts will be made to prepare wort and beer based on the analysed raw material.

## Figures and Tables

**Table 1:** Qualitative characteristics of hemp seeds.

Parameter	1000-Seed Weight	Moisture	Grading by Size					Seeds Viability	Water Sensitivity	Water Uptake Capacity	Appropriate Cross Section
			<1.5 mm	1.5 mm	1.8 mm	2.2 mm	2.5 mm				
	g	%									
Sample	11.76 ± 0.27	8.63 ± 0.23	6.90 ± 0.76	2.60 ± 0.32	36.53 ± 2.50	9.85 ± 2.40	43.83 ± 0.74	78.67 ± 4.16	58.02 ± 8.32	37.31 ± 0.84	92.97 ± 1.10

**Table 2:** Amount of total phytate and profile of inositol phosphates in hemp seeds (*Cannabis sativa* L.) subjected to malting.

Sample	Phytate (InsP_6_)		InsP_6_	InsP_5_	InsP_4_	InsP_3_	InsP_2-1_
	Total	Reduction	Total [% Relative Peak Area; Average]				
	[mg∙g^−1^]	[%]					
raw	56.25 ^b^	-	25.35	n.d.	n.d.	4.46	74.25
malted	45.69 ^a^	19	37.63	n.d.	n.d.	6.94	55.43

Notes: Values in columns are means ± SD of the sample; n = 3; values with different superscripts within columns are significantly different (*p* < 0.05).

**Table 3:** Gradient elution program for inositol phosphates analysis.

Time (min)	Mobile Phase A (%)	Mobile Phase B (%)
0.0	5	95
20.0	28	72
21.0	85	15
28.0	85	15
28.1	5	95
32.1	5	95

## Data Availability

Data are contained within the article.
